# Autonomic conflict exacerbates long QT associated ventricular arrhythmias

**DOI:** 10.1016/j.yjmcc.2018.02.001

**Published:** 2018-03

**Authors:** James Winter, Michael J. Tipton, Michael J. Shattock

**Affiliations:** a School of Cardiovascular Medicine and Sciences, King's College London, UK; b Extreme Environments Laboratory, Department of Sport & Exercise Science, University of Portsmouth, UK

**Keywords:** Autonomic conflict, Long QT syndrome, Torsades de pointes, Sympathetic, Parasympathetic, Autonomic nervous system, EAD, early afterdepolarisation, LQTS, long QT syndrome, TdP, torsades de pointes, TpTe, T-wave peak-to-end interval, VT, ventricular tachycardia, VNS, vagus nerve stimulation

## Abstract

This study tested the hypothesis that concomitant sympathetic and parasympathetic stimulation (“autonomic conflict”) may act as a trigger for arrhythmias in long QT syndrome (LQTS). Studies were performed in isolated innervated rabbit hearts treated with clofilium (100 nmol/L); a potassium channel blocker. The influence of vagus nerve stimulation (VNS) on spontaneous ventricular arrhythmias was assessed in the absence/presence of sustained noradrenaline perfusion (100 nmol/L) and with sudden adrenergic stress (injections of noradrenaline into the perfusion line). Hearts were instrumented for a pseudo-electrocardiogram and monophasic action potential recordings. VNS, which slows heart rate, was associated with a stimulation frequency-dependent incidence of spontaneous early after-depolarisations (EADs) and ventricular tachycardia (VT), best predicted by the duration of the electrocardiographic T-wave and by triangulation of the ventricular action potential. In the presence of sustained (steady-state) noradrenaline perfusion, the incidence of EADs and VT with VNS was decreased from 73/55% to 45/27%, respectively. However, sudden adrenergic stress, imposed during periods of sustained VNS, was associated with a transient increase in the incidence of severity of observed arrhythmias, as indicated by an increase in the average arrhythmias score (1.6 ± 0.4 vs. 2.1 ± 0.7, *p* = .01). Analysis of electrophysiological parameters suggests that sudden adrenergic stress is associated with a transient prolongation, and increased triangulation, of the ventricular action potential, which may predispose to triggered activity. This study demonstrates that autonomic conflict is a pro-arrhythmic stimulus in LQTS. However, combined adrenergic and parasympathetic stimulation has a complex relationship with arrhythmogenicity, with differences in the effects of steady-state adrenergic activation vs. sudden adrenergic stress.

## Highlights

• Long QT syndrome associated arrhythmias were studied with dual autonomic stimulation.
• EADs and TdP were triggered by increased vagal tone.
• Sudden adrenergic stress increased the severity of observed arrhythmias.
• Arrhythmias were prevented by sustained adrenergic stimulation.
• The sequence of autonomic stimulation influences the severity of long QT arrhythmias.

## 1 Introduction

Malignant ventricular arrhythmias (e.g. torsades de pointes - TdP) are the primary cause of syncope and premature death in patients with abnormal prolongation of the electrocardiographic QT interval. Despite extensive research, the role of the autonomic nervous system in long QT syndrome (LQTS) associated arrhythmogenesis is imperfectly understood. The conventional textbook picture of the cardiac autonomic control suggests the two branches of the autonomic nervous system exert reciprocal actions on the heart, such as in the baroreceptor reflex. In reality, this represents an over-simplification of a system in which simultaneous and synchronous activation often occurs [[1], [2], [3]]. For example, during cold-water immersion, activation of the parasympathetically mediated mammalian diving reflex and sympathetic/adrenergically driven cold-water shock response occurs simultaneously [[1], [2], [3]].

Cold-water submersion (head under) is known to be a trigger for supraventricular and ventricular arrhythmias in young, seemingly healthy people, who do not exhibit evidence of arrhythmias at rest [[3], [4], [5], [6], [7]]. These arrhythmic events are not commonly observed in the same individuals during head out cold-water immersions (sympathetic stimulation, no parasympathetic stimulations), or during facial immersion, with breath-hold, which activates the parasympathetic diving reflex and not the cold-shock (sympathetic) response. This suggests that simultaneous autonomic activation, as in whole-body cold-water submersion, might be an arrhythmogenic stimulus, and is supported by other anecdotal observations [1], but has never been tested experimentally. We recently hypothesised that the sudden and simultaneous sympathetic and parasympathetic activity, termed ‘autonomic conflict’, may be a cause of arrhythmias associated with cold-water submersion and more generally in situations where autonomic co-activation occurs in conjunction with existing predisposing factors [3].

Post-mortem analysis using DNA sequencing has revealed that nearly 30% of the victims of seemingly unexplained drowning have cardiac ion channel mutations [8]. Moreover, a strong association between swimming and arrhythmias/sudden cardiac death has been shown in children with heritable LQTS and swimming is recognised as a gene-specific trigger for TdP in patients with congenital LQTS type 1 [[9], [10], [11], [12]]. Could this represent something more than a simple increase in adrenergic tone? Similarly, patients with LQTS type 2 generally die during times of sudden stress, such as when awoken by an alarm clock, suggesting that it is sympathetic activation during periods of high parasympathetic tone that triggers TdP [10,13]. Evidence from 24-hour electrocardiogram recordings in patients with acquired LQTS indicates that while prevailing rates are relatively slow, there is a significant increase in heart rate in the minutes preceding electrical instability and TdP [14]. Therefore, we propose that the sequence and timing of autonomic activation plays a critical role in the induction of LQTS associated arrhythmias. In this study, we examined the role of autonomic conflict as a trigger for ventricular arrhythmias in conditions of acquired (type 2) LQTS. We hypothesised that concomitant sympathetic and parasympathetic stimulation would act as a trigger for LQTS associated arrhythmogenesis.

## 2 Methods

### 2.1 Animal welfare

All procedures were undertaken in accordance with ethical guidelines set out by the UK Animals (Scientific Procedures) Act 1986 and Directive 2010/63/EU of the European Parliament on the protection of animals used for scientific purposes. Studies conformed the Guide for the Care and Use of Laboratory Animals published by the U.S. National Institutes of Health under assurance number A5634-01. Studies were approved by local ethics review at King's College London.

### 2.2 Isolated innervated rabbit heart

All studies utilised the isolated innervated rabbit heart as described by Ng et al., with minor modifications [10]. Experiments were performed in New Zealand White Rabbits (2.5–4.5 kg). Animals were pre-sedated using a mixture of ketamine (Ketaset, 10 mg/kg, Fort Dodge, UK), medetomidine hydrochloride (Sedator, 0.2 mg/kg, Dechra, UK) and butorphanol (Torbugesic, 0.05 mg/kg, Fort Dodge, UK) (i.m.). Anaesthesia was induced and maintained using propofol (5 mg as required, Rapinovet, Schering-Plough Animal Health, UK), with concomitant heparin (1000 units, Multiparin, UK). Animals were intubated and ventilated at 60 breaths per minute of room air. The major blood vessels leading to and from the thorax were ligated and cut. Animals were killed by an overdose of sodium pentobarbitone solution (160 mg/kg i.v.). The descending aorta was cannulated and the preparation excised from C1-T12 (on ice). Hearts were retrogradely perfused with an oxygenated Krebs-Hensleit buffer, containing (in mM): NaCl 114, KCl 4, CaCl 1.8, NaHCO_3_ 24, MgSO_4_ 1, NaH_2_PO_4_ 1.1, glucose 11.0 and sodium pyruvate 1.0. Perfusion rate was set to give an aortic pressure of 70–80 mmHg. The right vagus nerve was isolated from the surrounding tissues and placed on silver bipolar stimulating electrodes connected to a constant voltage stimulator (DS2A, Digitimer, UK).

### 2.3 Experimental protocols

All hearts were instrumented for recording of a pseudo bipolar electrocardiogram (low pass = 100 Hz, high pass = 0.3 Hz, sampling = 1KHz) and monophasic action potential from the left ventricular free wall (low pass = 100 Hz, high pass = 0.3 Hz, sampling = 1 kHz). Hearts were perfused with clofilium tosylate (100 nmol/L in DMSO) for 20-min prior to the commencement of the experimental protocols and throughout the study. 100 nmol/L clofilium was sufficient to cause substantive QT prolongation without AV-block, which can occur with higher concentrations of class III anti-arrhythmic agents.

#### 2.3.1 Protocol 1 (sustained noradrenaline)

Cardiac responses to VNS were assessed at 2, 5, 10 and 20 Hz stimulation [pulse width = 1 ms, amplitude = 10 V, duration = 2 min per frequency] in the absence and presence of 100 nmol/L noradrenaline (+50 μM ascorbate). This represents a submaximal concentration in the rabbit heart and produces a marked and sustained chronotropic and electrophysiological action. Noradrenaline was perfused for a minimum of 10-min prior to nerve stimulation.

#### 2.3.2 Protocol 2 (bolus noradrenaline)

Noradrenaline (0.1 mL of 1 mM stock) was injected directly into the bubble trap of the perfusion apparatus at intrinsic heart rates and following 2-min of VNS at 5 and 10 Hz. Hearts were allowed to recover for 10-min between injections.

### 2.4 Blinding and randomisation

To reduced inter-operator variability, studies were conducted and analysed by a single experimenter. The sequence of experimental protocols and order of stimulations in Protocols 1 and 2 were randomised prior to the study. The nature of the paired study design prevented blinding of the experimenter to the study protocols.

### 2.5 Data analysis

Experiments were ranked per the severity of observed arrhythmias, using a predefined arrhythmias score (no arrhythmias = 1, early afterdepolarisations (EADs) = 2, ventricular tachycardia (VT) = 3, sustained VT (>20 s) or ventricular fibrillation = 4). Note that this is a simple hierarchical scoring system, in which the absolute score at each level is not a true quantification of arrhythmias severity. Curtis and Walker have previously shown the validity of such hierarchical systems for use in arrhythmias quantification [15]. EADs were assessed first in monophasic action potential recordings and then confirmed in ECG recordings - evidenced by an R-T ectopic in the latter and a premature beat occurring before repolarisation of the preceding action potential in MAP recordings. Because TdP can present monomorphic or polymorphic patterns in ECG recordings from the same subject, depending on the relative angle of the recording electrode, presented values are the number of episodes of VT. In most cases, ECG and monophasic action potential parameters were assessed from 10-beat averages of normally conducted beats. Monophasic action potential duration was assessed at 90% repolarisation (MAPD_90_). Action potential triangulation was calculated as MAPD_90_-MAPD_30_.

### 2.6 Statistics

Arrhythmias scores were compared by Wilcoxon's matched-groups signed rank-sum tests. For electrophysiological parameters, parametric statistical analyses were applied on the basis that the distribution of the QT interval is Gaussian and on the assumption that other electrogram parameters and related measures (e.g. action potential triangulation) are similarly distributed. Statistical comparisons were made using paired Student's *t*-tests and repeated measures one or two-way ANOVA with Sidak's post-hoc tests, as appropriate. *p* < .05 was considered significant. Continuous variables are presented as means (standard deviation (SD)).

## 3 Results

### 3.1 VNS is a trigger for EADs and VT in LQTS

**Fig. 1:**
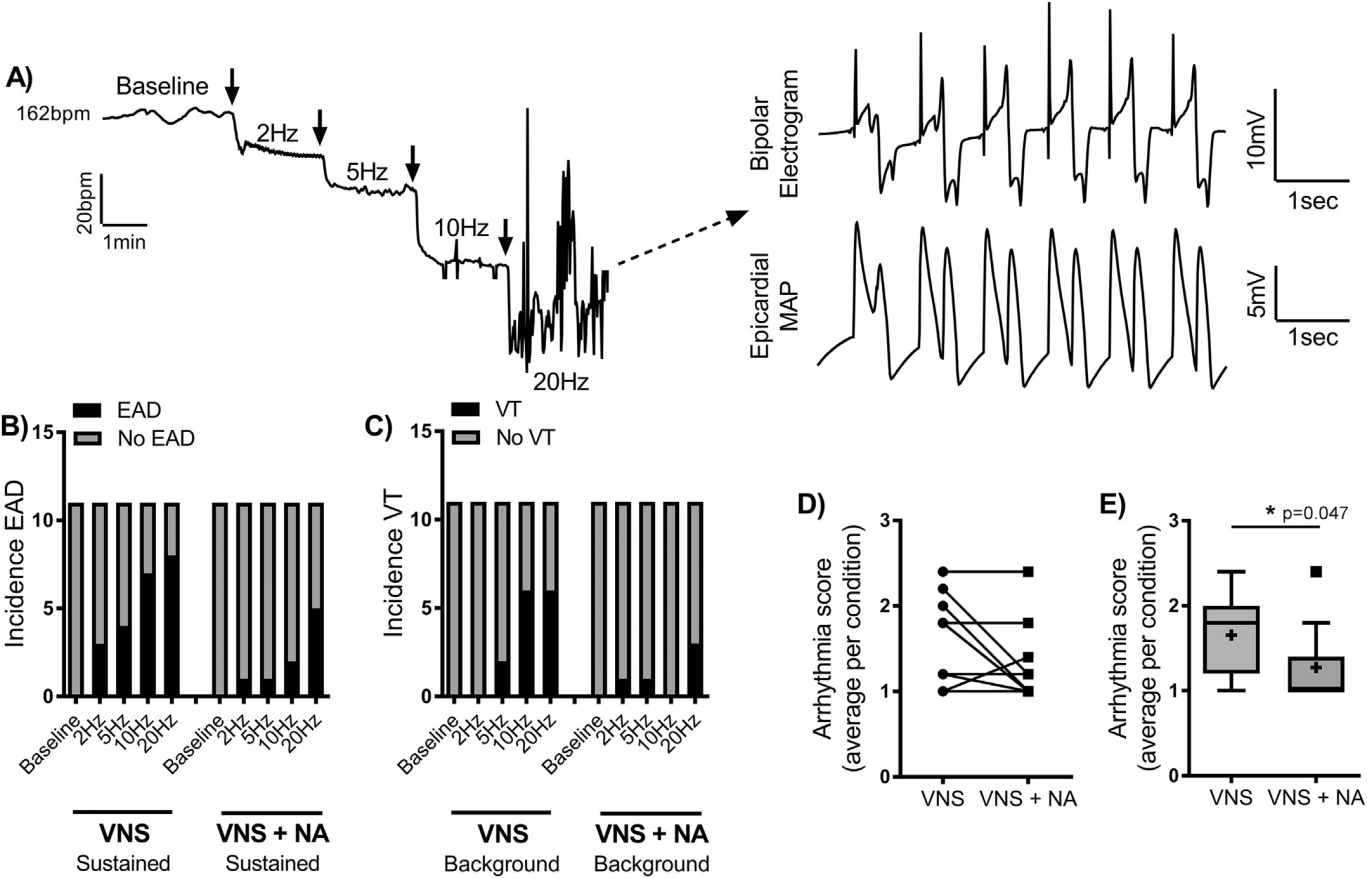
Sustained noradrenaline perfusion suppresses LQTS associated arrhythmias. A) Representative traces demonstrating the slowing of heart rate with increasing intensities of vagus nerve stimulation (VNS) in a clofilium-treated (100 nmol/L) rabbit heart. In this example, early afterdepolarisations (EADs) are triggered at 20 Hz VNS. B&C) Arrhythmias counts for 11-experiments demonstrating a reduction in the incidence of EADs and ventricular tachycardia (VT) with sustained noradrenaline (NA, 100 nmol/L) perfusion. D&E) Individual and average (Tukey's box plot) arrhythmias scores during VNS and with VNS + NA (sustained). Different from VNS alone; *p < .05. Comparisons by Wilcoxon's matched-groups signed rank-sum test (*n* = 11 hearts).

**Fig. 2:**
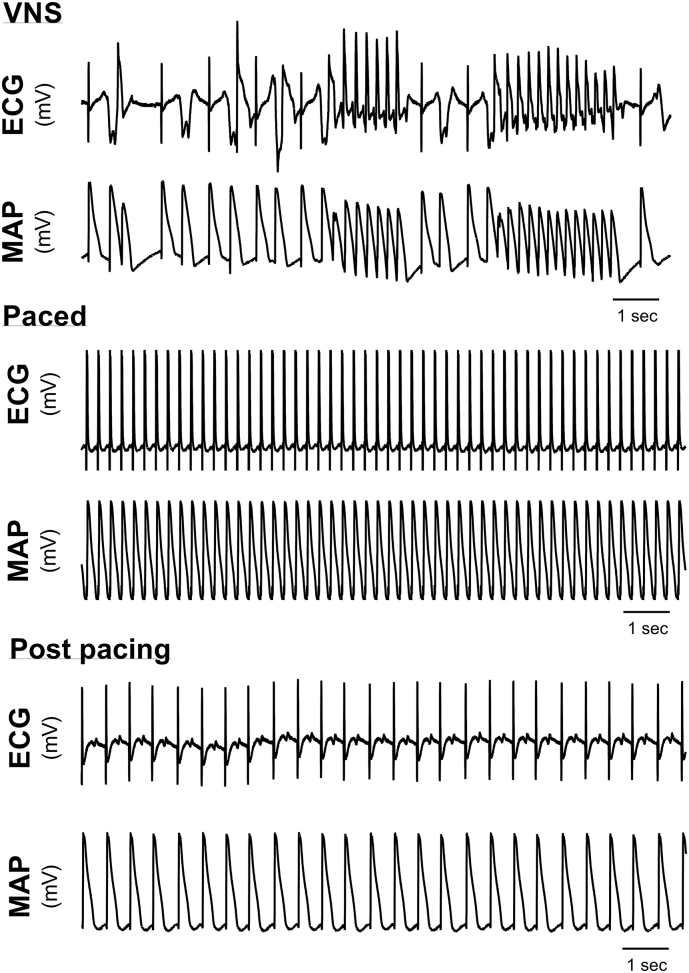
Suppression of VNS triggered arrhythmias by rapid pacing. Representative traces showing spontaneous episodes of early afterdepolarisations and ventricular tachycardia following vagus nerve stimulation (VNS), with subsequent rapid ventricular pacing (cycle length = 190 ms) and on the cessation of pacing (following 1-min of sustained pacing).

The effects of combined cloflium-treatment and VNS on the incidence of spontaneous EAD and VT in rabbit hearts are presented in Fig. 1. Perfusion of clofilium (100 nmol/L) caused a marked slowing of intrinsic heart rate from 203 ± 33 to 164 ± 29 beats per minute (*p* < .001) and prolongation of the QT interval (270 ± 34 vs. 177 ± 4 ms, *p* < .001), but was not associated with spontaneous ventricular arrhythmias. Meanwhile, EADs and TdP were readily triggered by electrical stimulation of the vagus nerve in clofilium treated rabbit hearts. This can be seen in the representative trace in Fig. 1A, showing a stimulation frequency-dependent slowing of heart rate and the triggering of EADs with high intensity stimulation (20 Hz). VNS did not induce ventricular arrhythmias in the absence of cloflium (data not shown). Data presented on the left of Fig. 1B&C summarise the incidence of arrhythmias with VNS in 11 experiments, and show that VNS acted as a stimulation frequency-dependent trigger for spontaneous EADs and VT in LQTS; with no arrhythmias observed in baseline conditions (i.e. in hearts beating at their intrinsic rate). The percentage of hearts exhibiting EADs increased from 0% at baseline to 73% with 20 Hz VNS, and the maximum incidence of spontaneous VT was 55%. Neither EADs or spontaneous VT were observed with VNS before clofilium perfusion (data not shown). Arrhythmias were suppressed by the restoration of normal beating rates, either spontaneously or by short-term ventricular pacing (after which hearts returned to sinus rhythm) (see Fig. 2). The pro-arrhythmic action of VNS is most likely attributed to changes in rate, and not due to direct effect on the ventricles, as VNS-triggered EADs and VT were abolished by short term ventricular pacing (~1 min, Fig. 2). Furthermore, VNS mediated QT prolongation was completely abolished by ventricular pacing (see Supplementary Fig. 1), indicating that changes in rate are essential to the ventricular action of the vagus nerve in the rabbit heart (data from untreated hearts).

### 3.2 T-wave duration and action potential triangulation predict EADs during VNS

Table 1 presents a comparison of electrophysiological parameters in hearts with and without spontaneous EADs during VNS (5 Hz) and shows that the duration of the T-wave (TpTe interval), a measure of ventricular dispersion of repolarisation, and triangulation of the ventricular action potential, are the best predictors of VNS-associated triggered activity. EADs were not predicted by the RR interval, QT interval, APD and beat-to-beat variability in APD (as assessed from 10 sequential beats immediately before the first EAD or the last 10-beats before the cessation of VNS).

**Table 1:** Electrophysiological predictors of early afterdepolarisations.

Parameters	Experiments without EADs	Experiments with EADs	*p*-value
RR interval (ms)	465 (98)	560 (105)	0.139
QT Interval (ms)	335 (38)	382 (50)	0.098
TpTe Interval (ms)	59 (10)	99 (21)	*0.001
APD_90_ (ms)	261 (26)	285 (42)	0.176
APD BBV (ms)	2.8 (3.9)	12.3 (24.0)	0.364
Action potential triangulation (ms)	125 (17)	164 (39)	*0.045

Comparison of electrocardiogram and monophasic action potential parameters in hearts with and without early afterdepolarisations (EADs) during vagus nerve stimulation (VNS) at 5 Hz. 10-beat averages immediately before the first EAD or the last 10-beats before the cessation of VNS. BBV = beat-to-beat variability. Data represent means (SD). Comparisons by unpaired Student's *t*-tests. *p < .05 (*n* = 6 per group).

### 3.3 Sustained noradrenaline perfusion suppresses long QT associated arrhythmias

In the presence of sustained (steady-state) noradrenaline, the propensity for EADs and VT with VNS was substantially reduced (Fig. 1B&C). With VNS at 20 Hz, 73% of hearts exhibited EADs in control conditions, compared to only 45% in the presence of noradrenaline. TdP exhibited a similar pattern, with an incidence of 55% in control conditions vs. 27% during noradrenaline perfusion. The average arrhythmias score, a measure of the severity of observed arrhythmias, was reduced by sustained noradrenaline perfusion (Fig. 1D&E).

### 3.4 VNS antagonises the action of catecholamines on heart rate but not on ventricular electrophysiology

**Fig. 3:**
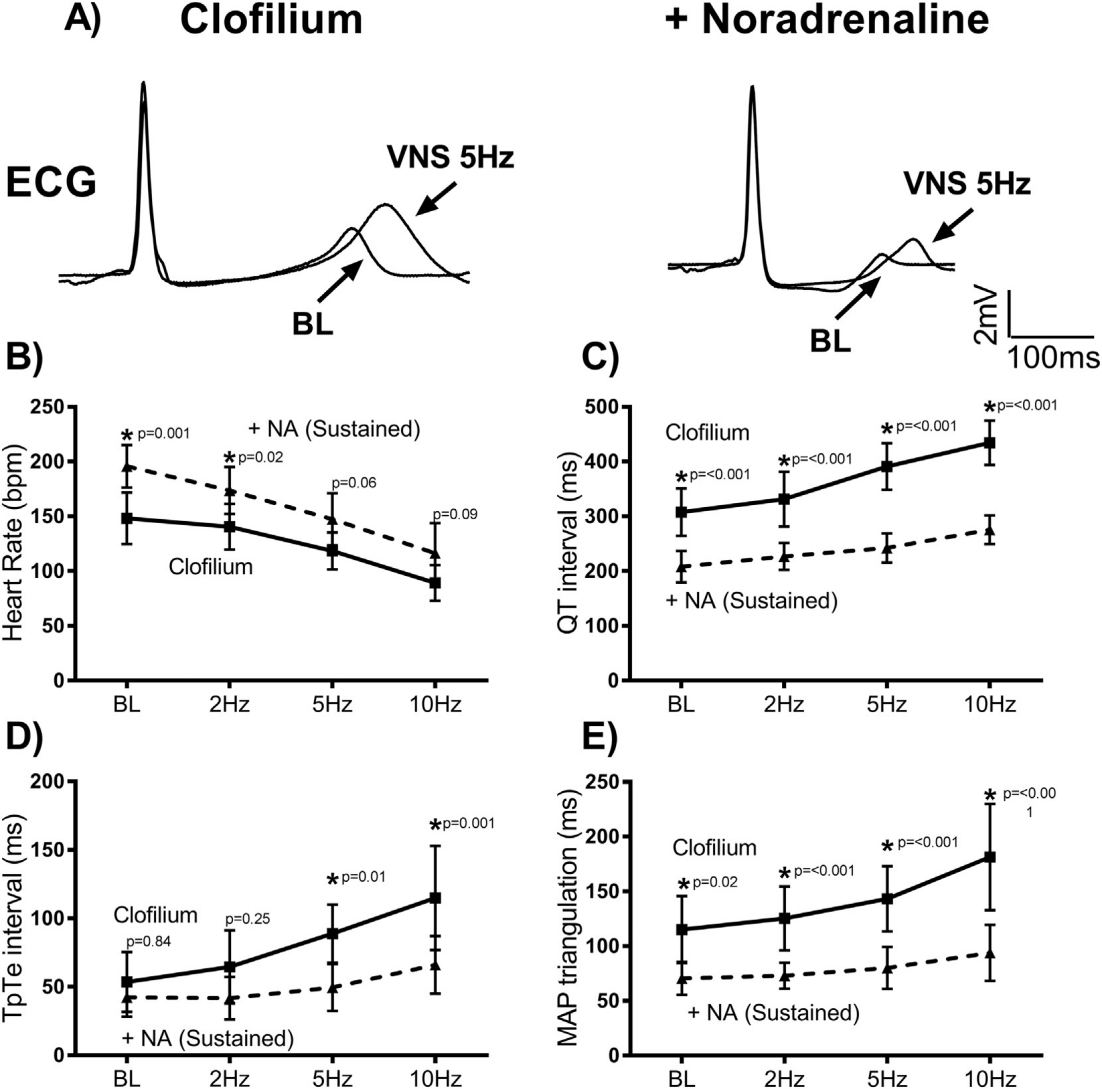
Electrophysiological changes during VNS with and without sustained noradrenaline. A) Representative traces demonstrating changes in electrocardiogram (ECG) parameters during VNS in a clofilium-treated (100 nmol/L) rabbit heart, with and without sustained noradrenaline (NA, 100 nmol/L) perfusion. B–E) Average values on the change in heart rate, QT interval, T-wave peak to end interval (TpTe) and monophasic action potential (MAP) triangulation. Data represent mean (SD). Different from clofilium (control); *p < .05. Two-way repeated measures ANOVA, with Sidak's post-hoc tests (*n* = 7 hearts).

The electrophysiological consequences of sustained (steady-state) noradrenaline perfusion are presented in Fig. 3. As expected, noradrenaline perfusion resulted in an increase in heart rate and shortening of the QT interval, as well as a reduction in the magnitude of action potential triangulation. In the presence of noradrenaline, VNS slowed heart rate to a greater degree than that observed in control conditions. At baseline, and with low intensity VNS (2 Hz), heart rate was elevated by noradrenaline, but this effect was lost with at higher intensities of stimulation (Fig. 3B) - showing that the vagus nerve antagonises the action of noradrenaline on heart rate. By comparison, adrenergic-dependent shortening of the QT interval was not reversed by VNS (Fig. 3C). In fact, the magnitude of VNS-dependent QT prolongation was less in the presence of catecholamines (68 ± 5 ms vs. 127 ± 14 ms, *p* = .003) and thus QT interval was substantially shorter at all frequencies of nerve stimulation, despite a similar underlying heart rate during high intensity VNS (see Fig. 3C). Similarly, a greater increase in ventricular dispersion of repolarisation (as indicated by prolongation of the TpTe interval) and triangulation of the action potential was observed with VNS in the absence of adrenergic stimulation (Fig. 3D&E). The TpTe interval increased by 136 ± 34% with VNS alone and 64 ± 22% with VNS and noradrenaline perfusion (*p* = .004). Action potential triangulation increased by 70 ± 16 ms with VNS in control conditions vs. 28 ± 9 ms with VNS in the presence noradrenaline (*p* = .003). Taken together, this suggests that VNS does not antagonise the electrophysiological action of exogenous noradrenaline in the rabbit ventricle, whilst accentuated antagonism is evident in changes in heart rate. This likely reflects the greater influence of the parasympathetic nervous system at the sinoatrial node vs. the ventricular myocardium. The anti-arrhythmic action of sustained noradrenaline perfusion in LQTS may be attributed to the relative shorter ventricular action potential and reduced action potential triangulation during combined autonomic stimulation, which prevents EADs. This action may be mediated by rate-dependent or rate-independent mechanisms, whereby the action potential intrinsically shortens at faster beating rates (a consequence of the kinetic properties of several ion channels and alterations in the concentration of intracellular Ca and Na ions), and because of increased outward current via the slow delayed rectifying K currents (via protein kinase A-dependent phosphorylation of the channel subunits). Our data suggest that rate-independent shortening of the ventricular action potential is the major mechanism mediating the anti-arrhythmic action of sustained noradrenaline perfusion, because with high intensity VNS there was no difference* in heart rate between VNS alone and VNS plus noradrenaline (Fig. 3B), but QT interval and APD were substantially shorter with combined stimulation at all levels of VNS (Fig. 3C). [*A trend is observed, possibly indicating a lack of statistical power to detect small differences between groups.]

### 3.5 Bolus noradrenaline facilitates and exacerbates arrhythmias associated with QT prolongation

Electrical pickup on the ECG recordings during direct sympathetic nerve stimulation prevents accurate assessment of arrhythmias in the isolated innervated rabbit heart. However, rapid infusion of noradrenaline appears to be a good correlate of sympathetic nerve stimulation. Supplementary Fig. 2 shows the heart rate response to bolus noradrenaline compared with moderate intensity bi-lateral sympathetic nerve stimulation (10 Hz, 40 V, 2 ms pulse duration). The presented data show that the initial heart rate response to noradrenaline is marginally slower than that of direct nerve stimulation (owing to perfusion delay), however, the maximum heart rate and time to peak heart rate response was similar between conditions.

**Fig. 4:**
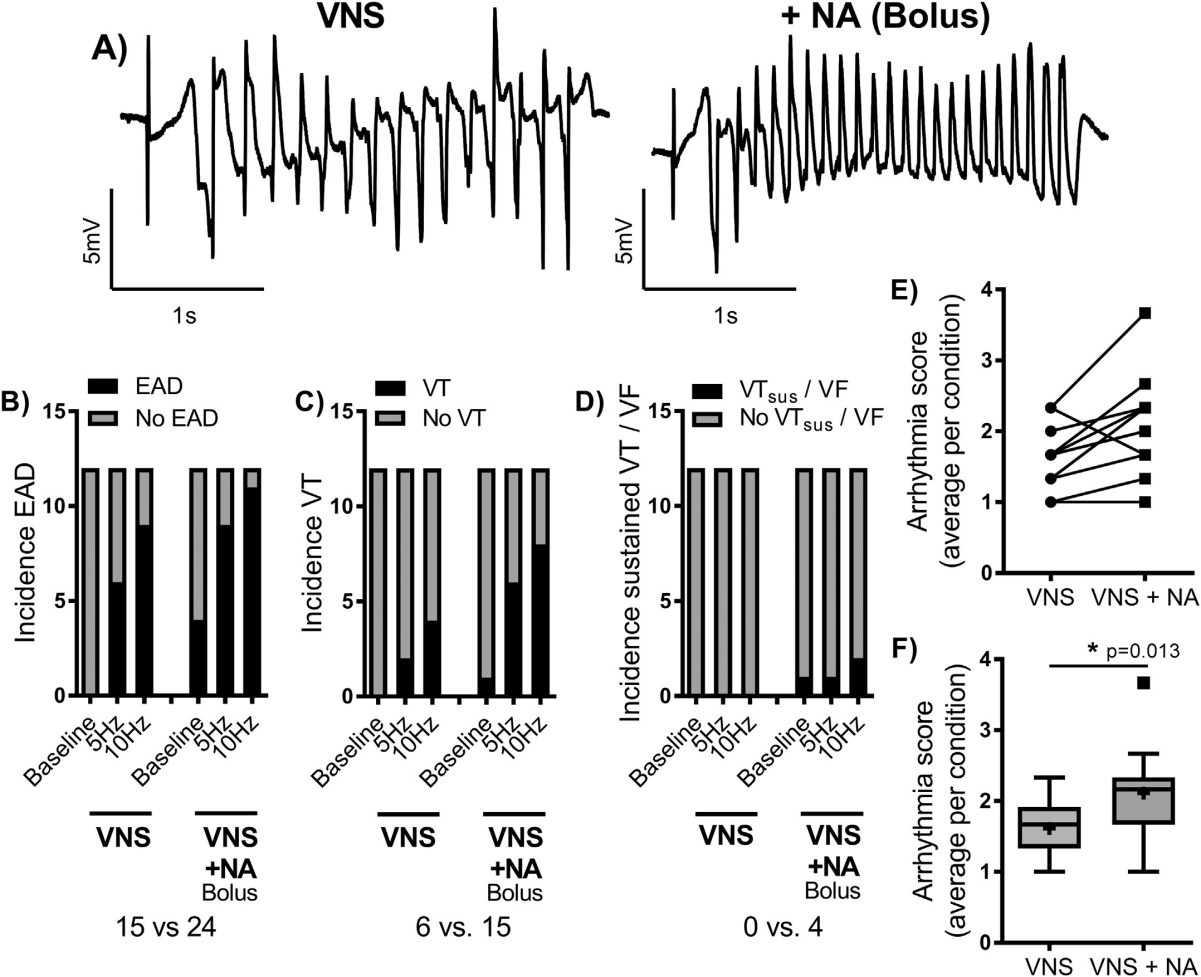
Bolus noradrenaline facilitates LQTS associated arrhythmias. A) Representative episodes of spontaneous ventricular tachycardia (VT) in a clofilium-treated (100 nmol/L) rabbit heart. The left trace was observed during VNS and the right following bolus noradrenaline (NA) perfusion (0.1 mL of 1 mM stock), in separate experiments. B-D) Arrhythmias counts for 12-experiments, demonstrating an increase in the incidence of early aftedepolarizations (EADs), VT and sustained VT (VT_sus_)/ventricular fibrillation (VF) with VNS + NA (bolus). E&F) Individual and average arrhythmias scores during VNS and with VNS + NA (bolus). Different from VNS alone; *p < .05. Comparisons by Wilcoxon's matched-groups signed rank-sum test (*n* = 12 hearts).

Data from 12 experiments on the influence of sudden adrenergic stress on the incidence of LQTS associated arrhythmias are presented in Fig. 4. Injection of a bolus of noradrenaline into the perfusion line acted in a bi-phasic manner- first increasing the risk of arrhythmias in the 10 to 20 second period immediately after injection and then acting to accelerate the underlying heart rate, suppressing arrhythmic events. In stage 1, bolus noradrenaline acted to increase the severity of the observed arrhythmias, with a dependence on the underlying conditions. For instance, noradrenaline commonly triggered EADs in the absence of pre-existing arrhythmias or caused VT in hearts already exhibiting ectopic activity. As such, VT was more commonly observed with noradrenaline perfusion during high intensities of VNS. In two of twelve hearts, bolus noradrenaline led to periods of sustained VT (>20 s), which in one case degenerated into ventricular fibrillation (VF). The incidence of spontaneous EADs, VT and sustained VT/VF with VNS and VNS plus bolus noradrenaline are presented in Fig. 4B–D. 67% of hearts exhibited spontaneous VT with VNS + bolus noradrenaline vs. 33% with VNS alone. Spontaneous EADs were observed in just 1 heart following noradrenaline perfusion in the absence of VNS. The severity of arrhythmic events was increased by combined stimulation, over VNS alone, as evidenced by an increase in the average arrhythmias score (Fig. 4E&F).

### 3.6 Bolus noradrenaline causes a transient increase in action potential duration and triangulation

**Fig. 5:**
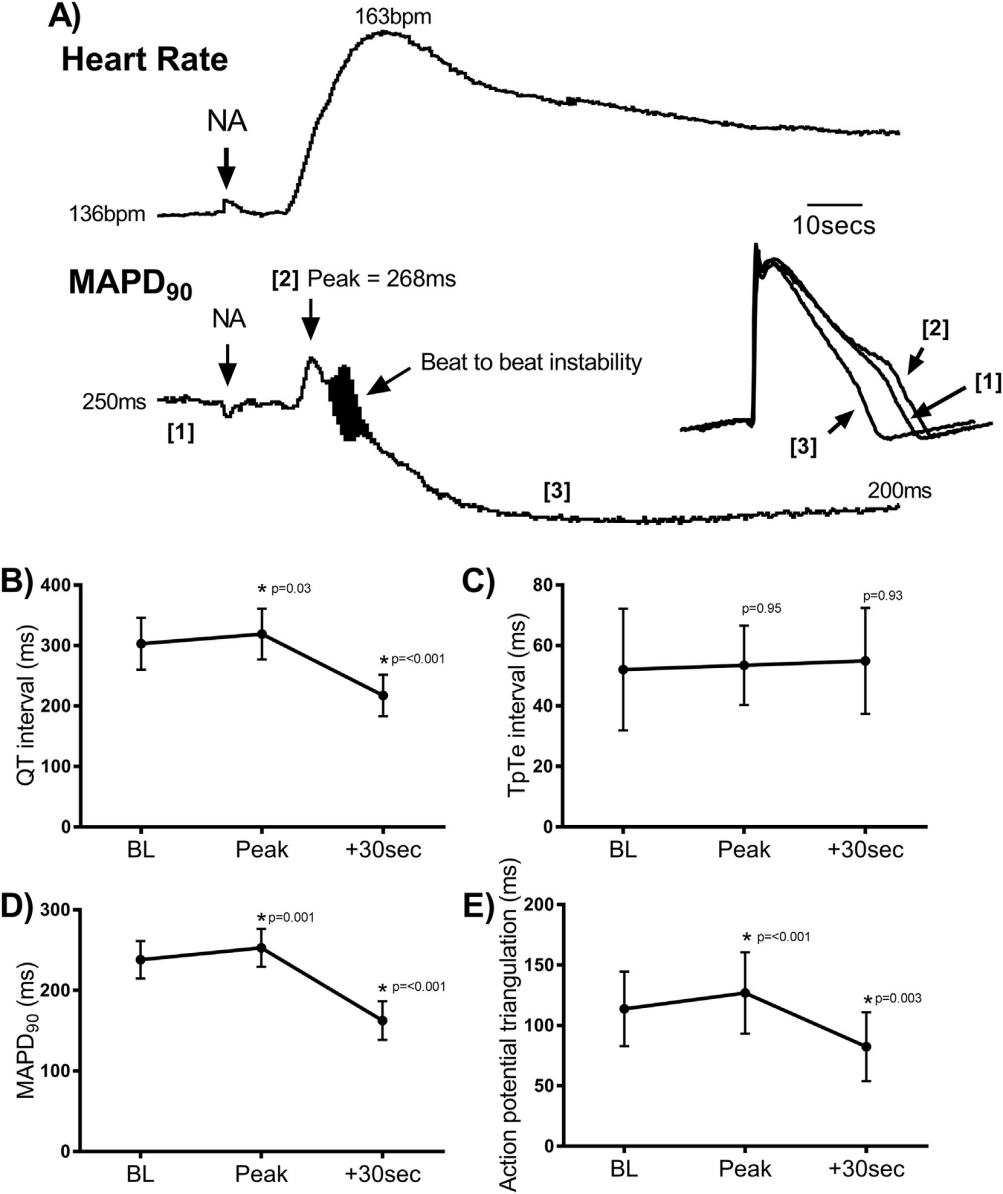
Electrophysiological effects of bolus noradrenaline in LQTS. A) Experimental trace demonstrating the change in heart rate and monophasic action potential duration (MAPD_90_) following bolus noradrenaline (NA) perfusion (0.1 mL of 1 mM stock) in a clofilium treated (100 nmol/L) rabbit heart. A transient increase in MAPD_90_ is seen immediately following NA perfusion. The inset shows representative action potentials recorded at the times indicated on the MAPD_90_ trace. B–E) Average values for QT interval, T-wave peak to end interval (TpTe), MAPD_90_ and action potential triangulation at baseline, at peak response and at 30-second post-peak response. Data represent mean (SD). Different from baseline; *p < .05. Comparisons by one-way ANOVA, with Sidak's post-hoc tests (*n* = 9 hearts).

To investigate the impact of sudden adrenergic stress on ventricular electrophysiology in LQTS we analysed changes in electrophysiological parameters following the injection of a bolus of noradrenaline in baseline conditions (i.e. in the absence of VNS). This was necessitated by the high incidence of arrhythmias during combined autonomic stimulation. These data are presented in Fig. 5. Bolus noradrenaline caused a rapid increase in heart rate that recovered to baseline over a 10-minute period (Fig. 5A). A transient increase in the duration of the QT interval, ventricular MAPD_90_ and magnitude of action potential triangulation was observed within approximately 10-s of noradrenaline injection; followed by a sustained shortening of ventricular repolarisation and reduction of action potential triangulation (Fig. 5B–D). This suggests that application of sudden adrenergic stress in LQTS leads to transient pro-arrhythmogenic alterations in ventricular electrophysiology. Indeed, the time course of changes in ventricular repolarisation paralleled the increase in the risk of arrhythmic events immediately following noradrenaline perfusion (during VNS), as well as the suppression of said arrhythmias with sustained adrenergic activation. Dispersion of repolarisation, as assessed from the duration of the TpTe interval, was similar at all time points (Fig. 5E). No overall difference in beat-to-beat variability for QT interval or APD was observed at any time point (data not shown).

## 4 Discussion

To our knowledge, this is the first study to show the importance of dynamic interplay between the two branches of the autonomic nervous system in LQTS-associated arrhythmogenesis. Our results show that combined adrenergic and parasympathetic stimulation has a complex relationship with arrhythmogenicity in LQTS. Whilst sustained (steady-state) noradrenaline perfusion suppresses the pro-arrhythmic action of VNS, application of a sudden adrenergic stressor during a period of sustained parasympathetic activation acts to increase the severity of observed arrhythmias. Noradrenaline rarely triggers spontaneous VT (1/12 hearts) in the absence of VNS, suggesting that concomitant autonomic activation (or ‘autonomic conflict’) is required for the adrenergic facilitation of LQTS-associated arrhythmias. Importantly, this observation parallels the clinical profile for patients with congenital LQTS type 2, who commonly die when woken suddenly by an alarm clock - suggesting that they experience lethal arrhythmias when a surge in sympathetic/adrenergic activity is superimposed during a time of high parasympathetic tone (i.e. during rest or sleep) [10,13]. By recapitulating LQTS type II using drugs that block I_Kr_, we show that the number and severity of arrhythmias observed with VNS alone is exacerbated by application of adrenergic stressor during sustained parasympathetic stimulation. Thus, the facilitation of arrhythmogenesis in LQTS by adrenergic stress is critically dependent upon the timing and sequence of autonomic stimulation. This dependence on the sequence of events likely contributes to the stochastic nature of arrhythmias and the difficulties in predicting any individual's risk of sudden death or the timing of that death.

Most of studies on the influence on adrenergic activation on cardiac electrophysiology have focussed on steady-state responses and there has been only limited investigation into the importance of dynamic changes in autonomic tone in LQTS. In studies in the isolated ventricular wedge preparation, Schimizu et al. reported that in LQTS type 2, noradrenaline perfusion leads to a transient increase in transmural dispersion of repolarisation [16]. The authors showed that increased repolarisation heterogeneity correlated with an increase in the susceptibility to spontaneous and electrically induced VT. In our analysis of T-wave duration we found no evidence that adrenergic stress results in an increase in dispersion of repolarisation in the rabbit ventricles, but observed a transient increase in action potential duration and triangulation. The rate of repolarisation is thought to be an important factor in the generation of EADs, as this may lead to the reactivation of the inward calcium current and the reversal of membrane potential - either directly via inward current through the L-type calcium channels or due to stimulated release of calcium from the sarcoplasmic reticulum [[17], [18], [19]]. Thus, the increase in action potential triangulation with sudden adrenergic stress combined with parasympathetic activation may facilitate EADs and explain the transient increase in risk of arrhythmias with concomitant autonomic stimulation. Notably, recurrent ectopic activity is one mechanism thought to underpin *TdP* and is a feasible mechanism for the increased incidence of VT observed during bolus noradrenaline injections [20]. Our observations parallel a report by Liu et al., who demonstrated a transient risk for EADs in isolated myocytes from rabbits with congenital LQTS type 2 on exposure to isoprenaline - attributed to differential kinetics for activation of the L-type calcium current and slow component of the delayed rectifying potassium current [21]. A similar kinetic mismatch in the targets for protein kinase A dependent phosphorylation was predicted by integrated computational modelling of calcium and beta-adrenergic signalling [22]. Interestingly, in a follow up study, Xie et al. predicted that that sudden adrenergic stress favours the transition from VT to VF, due to dynamic changes in APD restitution, which may have relevance to our observation that bolus noradrenaline is associated with a transient risk of more severe tachyarrhythmia [23]. Faster onset kinetics of the L-type calcium current may explain the transient increase in action potential duration, triangulation and incidence of VT with bolus noradrenaline perfusion in our study. With more sustained adrenergic stimulation, the current carried by I_Ks_ is increased, acting to shorten the action potential and to reduce the risk of further ectopic activity. The same mechanism could explain why sustained adrenergic stimulation acts to suppress arrhythmias. Indeed, loss-of-function mutations in the pore-forming subunits of channels that conduct I_Ks_ are the most common form of congenital LQTS. In this scenario, I_Ks_ is weaker and does not counteract the increase in L-type current during adrenergic-stimulation, promoting EADs. Another factor in the suppression of LQTS-associated arrhythmias by sustained adrenergic stimulation is the resulting acceleration of heart rate, which shortens the action potential, secondary to changes in the concentration of intracellular ions, including, Na and Ca. Bradycardia is a well-established trigger for drug-induced LQTS-related arrhythmias and tachypacing can prevent sudden death both acutely and as a long-term therapy [24,25].

That VNS is an independent trigger for arrhythmias in drug-induced LQTS is not surprising and is in keeping with existing clinical and experimental evidence. Bradycardia is a well-established risk factor for TdP and Farkas et al. previously demonstrated that the induction of TdP in the phenylephrine-sensitised rabbit is dependent upon activation of the parasympathetic nervous system [26]. Phenylephrine, a selective alpha1-adrenoceptor agonist, increases blood pressure and activates the baroreceptor reflex, leading to a slowing of heart rate via the vagus nerve. In our study, VNS causes prolongation and triangulation of the ventricular action potential, which likely underpins the pro-arrhythmogenic action of parasympathetic activation in LQTS. It is notable that one of the best correlates of ectopic activity following VNS was the TpTe interval, a measure of dispersion of repolarisation. This observation could indicate the requirement for a critical gradient of repolarisation in order that ectopic beats can propagate through the ventricular myocardium. Alternatively, the TpTe interval may simply reflect the development of islands of tissue with prolonged APD from where ectopic activity first arises [27]. Furthermore, increased dispersion of repolarisation is a substrate that favours re-entrant arrhythmias and may predispose to TdP, in keeping with our previous observations [28].

Beta-adrenoceptor antagonists are the first line therapy for congenital LQTS, with an efficacy that is determined by the molecular basis of QT-prolongation. Beta-adrenoceptor blockade is reported to be most effective in LQTS type 1 and only moderately effective in LQTS type 2 and LQTS type 3 [10]. On this basis, we propose that the partial effectiveness of beta-blockade in congenital LQTS type 2 reflects that arrhythmias can be triggered by parasympathetic activation alone, but that the incidence and severity of arrhythmias is increased by sudden adrenergic stress. Beta-blockade likely inhibits the latter case, but clearly this does not entirely abolish the risk of TdP in this patient population. Many useful therapeutic drugs, including antipsychotics and antiarrhythmics, also cause QT prolongation and carry risk of TdP and sudden cardiac death [29]. Where the risk-to-benefit ratio is high, QT-prolonging drugs should be withdrawn in patients exhibiting excessive QT prolongation or symptoms of LQTS (e.g. dizziness, syncope). However, when the risk to benefit ratio is low (i.e. low-risk, high-benefit), our data suggest that patients could benefit from prophylactic beta-adrenoceptor blockade. That is to say, beta-blockade may be an effective strategy to mitigate risk of sudden death in drug-induced LQTS, in addition to congenital forms, and where there is no alternative to use of QT-prolonging drugs. Symptoms of syncope and the risk of sudden death in patients with congenital LQTS who are refractory to beta-blockade can also be mitigated by left sympathetic denervation, a surgical procedure that involves ablation of left stellate ganglion; the major source of sympathetic efferent nerve fibers that innervate the ventricles. Schwartz et al. have previously shown the efficacy of this approach in patients with congenital LQTS who continue with symptoms of syncope or cardiac arrest despite beta-blocker therapy [30]. With type 2 LQTS, where bradycardia is an independent trigger for arrhythmias, further benefits could be provided by the combined use of beta-blockers/denervation and chronic atrial pacing [25].

We have previously suggested that autonomic conflict, the simultaneous activation of the sympathetic and parasympathetic inputs to the heart, might be an arrhythmogenic trigger [3]. The basis for this is observations of substantive cardiac dysrhythmia and arrhythmias in young healthy adults within the first minute of submersion in cold-water; a potent stimulator of the sympathetically driven cold-water shock response and parasympathetcally mediated mammalian diving reflex. Importantly, similar events are not commonly observed in the same participants during head out cold-water immersion, or with facial immersion alone, suggesting that autonomic co-activation plays a critical role. We recently reported that combined autonomic stimulation (i.e. sustained noradrenaline + VNS) is insufficient to cause significant arrhythmias in healthy isolated rabbit hearts [31]. However, we show presently that concomitant stimulation can trigger severe arrhythmias in the presence of a predisposing electrophysiological abnormality, and in a manner, that is critically dependent on the timing of the adrenergic stimulus. Autonomic conflict may also have broader importance in other forms of LQTS, for example, it may explain why swimming is a gene-specific trigger for TdP and sudden death in congenital LQTS type 1 [9]. Moreover, dynamic events, leading to sudden alterations in autonomic tone, are likely to be an important contributing factor in many arrhythmogenic syndromes and pre-disposing conditions. For example, swimming may be a trigger for arrhythmias in patients with genetic mutations associated with catecholinergic polymorphic ventricular tachycardia [11]. Greater understanding of how the dynamic changes in autonomic tone influence electrophysiology and arrhythmogensis in disease may be an important factor in the development of more effective therapeutic strategies for the management of sudden cardiac death.

Animal models of TdP are essential for studies of the arrhythmogenic processes in LQTS, and also have utility for safety screening of QT prolonging drugs, however, there are several limitations with existing experimental approaches. These include: the necessary use of anaesthetic agents in in vivo models [26], a failure to predict the pro-arrhythmic potential of recognised torsadegenic drugs [26], use of non-physiological conditions (e.g. perfusion of acetylcholine to mimic VNS), a reliance on expensive protocols (e.g. chronic atrioventricular block in the dog) [32], and the low incidence of TdP in the absence of electrical induction protocols [16]. In this study, we describe a novel protocol that recapitulates LQTS-associated arrhythmogenesis, including spontaneous EADs and VT. With concomitant autonomic stimulation (VNS + bolus noradrenaline) the maximum incidence of EADs and VT in our study was 92% and 67%, respectively. As such, the innervated rabbit heart may be a useful model for the study of LQTS-associated arrhythmias. This approach has several potential advantages over existing models, including; the preservation of natural cardiac activation and repolarisation patterns, the ability to perform repeat measurements in the same heart, no requirement for anaesthetic agents, that heart rate can be altered physiologically by direct nerve stimulation and great flexibility/adaptability (e.g. rapid washout of drugs, altered ionic composition). Moreover, the model is relatively low-cost and requires only minimal training to implement. This facility of the *ex vivo* innervated heart in drug safety screening will require extensive validation and is a focus for future work.

### 4.1 Limitations

There are several limitations that deserve discussion. Firstly, in preliminary experiments we found that direct sympathetic nerve stimulation caused significant electrical pickup on ECG recordings, making it difficult to accurately determine the onset and type of arrhythmias. For this reason, we used exogenously perfused noradrenaline to mimic the effects of nerve stimulation. For the same reason, we are unable to compare the electrophysiological responses to noradrenaline and SNS in our experimental model, and so cannot conclude as to how similar bolus noradrenaline is to the traditional “fight or flight” response, which is likely mediated by local neurotransmitter release. Further studies, utilising techniques such as optical mapping, which do not suffer from electrical interference, could be used to address this limitation and to dissect out the underlying cellular mechanisms.

Secondly, both ketamine and medetomidine can influence autonomic control. However, as experiments were performed in an in vitro preparation, with significant time for drug washout, we believe this is unlikely to influence our results. Moreover, nerve responses were robust and reproducible throughout the experimental protocols.

Thirdly, the incidence of VT at baseline between protocols 1 and 2 was modestly different (2 vs. 4 episodes), which could simply reflect that more extended periods of VNS (as in protocol 1) are associated with a higher incidence of spontaneous arrhythmias. However, given that difference between the groups is not statistically significant, this observation may simply be down to chance.

Fourthly, on the slowing of heart rate with VNS, it was common for map recordings to exhibit unstable diastolic potentials (when previously stable at baseline), which was likely attributed to slight movement of the recording electrode secondary to altered contractile motion of the heart. This can be seen in the example trace shown in Fig. 1A. To avoid mechanical triggering of arrhythmias, we opted not to re-orientate the electrode mid protocol. However, despite the observed instability, it was still possible to accurately detect the onset of EADs, particularly as our analysis relied on concomitant observations in MAP recordings and in the ECG. Fifthly, we employed unilateral right-sided VNS and so cannot conclude whether our findings also apply to left or bilateral stimulation protocols. However, previous studies indicate that there is no difference in the chronotropic and electrophysiological effects of left and right-sided VNS in pigs, which corroborates unpublished observations from our laboratory (in rabbits) [33].

Finally, clofilium is a K channel blocker with a similar IC_50_ to E4031 and dofetilide, and has proven efficacy for the induction of TdP in rabbits [34]. The purpose of the present study was to develop a model that replicates congenital LQTS and not necessarily to compare directly to clinically used (or historical) pharmaceutical agents (e.g. d-sotalol, dofetilide). Whilst alternative class III agents are more commonly employed in experimental studies, many K channel blockers exhibit similar potency and we do not believe that our use of clofilium detracts from the conclusions of the present study [34]. Moreover, we have previously shown that VNS can trigger TdP in rabbit hearts treated with E4031, a result replicated presently with clofilium [28].

## 5 Conclusions

Autonomic conflict, the simultaneous activation of parasympathetic and sympathetic autonomic inputs to the heart, can facilitate arrhythmogenesis in LQTS. *Dynamic* alterations in autonomic tone may be an important contributory factor in many arrhythmogenic syndromes or in conditions that predispose to ventricular arrhythmias.

## Funding

J.W. (FS/16/35/31952) and M.J.S. (RG/12/4/29426) are supported by the British Heart Foundation.

## Appendix A Supplementary data

Supplementary material Image 1
