# Supplementary material for: Autonomic conflict exacerbates long QT associated ventricular arrhythmias
Source: J Mol Cell Cardiol. 2018 Mar;116:145–54. doi: 10.1016/j.yjmcc.2018.02.001 (PMC5855091; doi:10.1016/j.yjmcc.2018.02.001)
Supplement: Supplementary file 1 — Supplementary material [file mmc1.docx]

**Supplementary material**

**“Autonomic conflict exacerbates long-QT associated ventricular arrhythmia”**


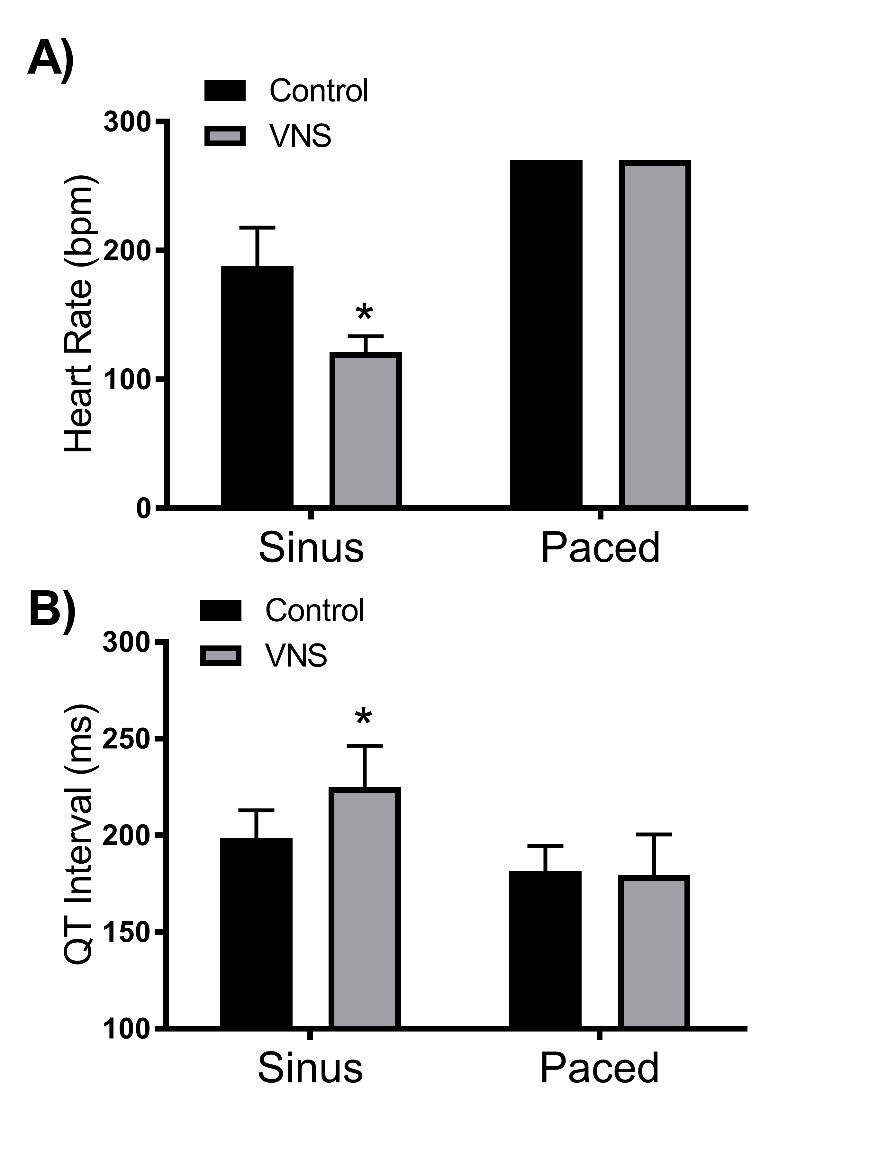


**Supplementary Figure 1.** A&B) Mean data from untreated rabbit hearts showing the effects of vagus nerve stimulation (VNS) on heart rate and QT interval in sinus rhythm and during constant ventricular pacing. Two-way repeated measures ANOVA, with Sidak’s post-hoc tests. The effects of VNS; *p<0.01. (n=4 hearts)


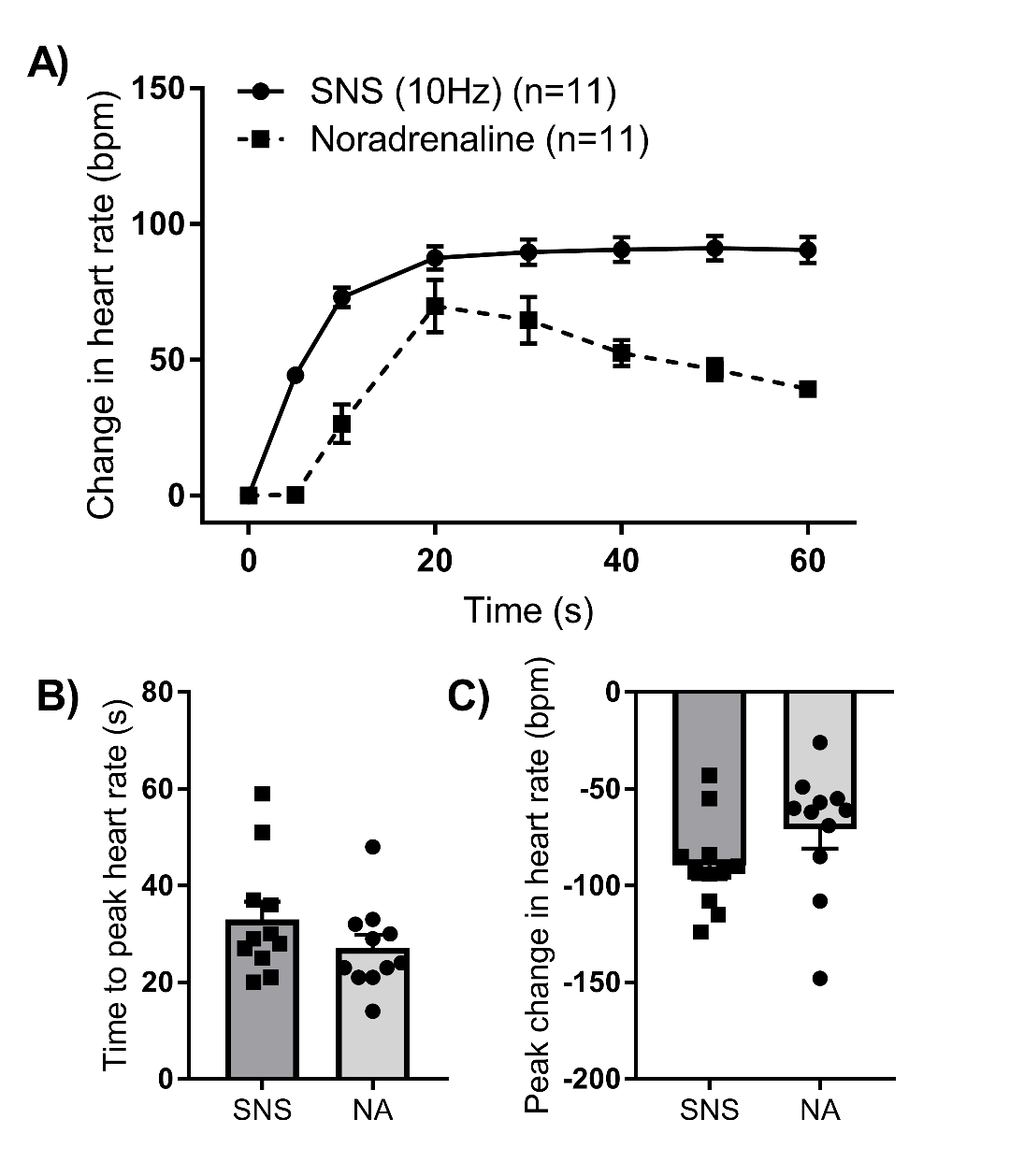


**Supplementary Figure 2. Comparison of heart rate response to sympathetic nerve stimulation and bolus noradrenaline injection.** A) Temporal profile of the change in heart rate with bilateral electrical stimulation of the cardiac sympathetic efferent nerves (10Hz, 2ms pulse width, 40V) and with injection of 0.1ml of 1mM noradrenaline (NA) stock solution into the perfusion line. B&C) Mean values on the time to peak heart rate responses and the change in heart rate with sympathetic nerve stimulation (SNS) and NA bolus injection.
